# Sex Differences in Cognitive Reflection: A Meta-Analysis

**DOI:** 10.3390/jintelligence12040039

**Published:** 2024-03-29

**Authors:** Inmaculada Otero, Alexandra Martínez, Dámaris Cuadrado, Mario Lado, Silvia Moscoso, Jesús F. Salgado

**Affiliations:** Faculty of Labour Relations, University of Santiago de Compostela, Campus Vida, 15782 Santiago de Compostela, Spain; alexandra.martinez@usc.es (A.M.); damaris.cuadrado@usc.es (D.C.); mario.lado@usc.es (M.L.); silvia.moscoso@usc.es (S.M.); jesus.salgado@usc.es (J.F.S.)

**Keywords:** cognitive reflection, sex differences, meta-analysis, cognitive reflection test

## Abstract

The current study presents a meta-analytic review of the differences between men and women in cognitive reflection (CR). The study also explores whether the type of CR test (i.e., numerical tests and verbal tests) moderates the relationship between CR and sex. The results showed that men score higher than women on CR, although the magnitude of these differences was small. We also found out that the type of CR test moderates the sex differences in CR, especially in the numerical tests. In addition, the results showed that the length of numerical tests (i.e., number of items) does not affect the differences between men and women in CR. Finally, the implications of these results are discussed, and future research is suggested.

## 1. Introduction

Human thinking is often characterized as an interaction between intuition and deliberation. Sometimes, a solution to a problem emerges in our mind quickly and without any effort. At other times, finding a robust solution will take time and elaborated thinking (De Neys 2017). These types of reasoning are usually known as Type 1 (intuitive) and Type 2 (deliberate) thinking, and this approach is explained by the Dual Process Theories (Evans and Stanovich 2013; Evans and Wason 1976; Wason and Evans 1975). According to this approach, Type 1 (T1) thinking produces quick, emotional, intuitive, impulsive, as well as associative responses. It works effortlessly, automatizing behaviors through learning and consistent experience with one’s environment (Kahneman and Frederick 2002; Logan 1988; Smith and DeCoster 2000). Type 2 (T2) thinking produces analytical, rational, deliberative, as well as rule-guided responses. It operates slowly, with effort and concentration, demanding cognitive resources (Kahneman and Frederick 2002). T1 thinking is often associated with the use of heuristics and shortcuts to be quick and to save cognitive resources when making judgments. In a parallel way, T2 thinking can still be biased, but it is hoped that true metacognition will see multiple sides of an issue to reduce bias (Epstein 2003; Kahneman and Frederick 2002, 2005; Sloman 1996; Stanovich 2009; Toplak et al. 2011, 2014).

People vary in their propensity to activate these types of thinking, although situations can also affect the use of T1 and T2 thinking. For example, experts can be more autonomous in their thinking, and situations, like emergencies, can rely on heuristics to get through such a situation. So, heuristics can be essential in some workplaces. The individual difference in activating these types of thinking has been labelled as cognitive reflection (CR), and it has been defined as the individual ability or disposition to stop the first impulsive response that our mind offers and to activate the reflective mechanisms that allow us to find an answer, make a decision, or carry out a specific behavior in a more thoughtful way (Frederick 2005; Kahneman and Frederick 2002). (Kahneman and Frederick 2002, 2005; Frederick 2005; Kahneman 2011) developed the most popular measure to assess CR, i.e., the cognitive reflection test (CRT). The CRT is a test composed of three arithmetical problems that trigger an immediate answer, although this immediate answer is usually erroneous. To correctly answer the items, individuals have to override their first response in favor of the alternative one, which is reflective, deliberative, and more cognitive elaborated. 

Although the 3-item CRT (also called CRT-3; Frederick 2005; Kahneman 2011; Kahneman and Frederick 2002, 2005) is the most popular test, other measures to assess the CR were developed as well. For instance, some researchers have created larger CR tests with new and different items (see, Salgado et al. 2019; Sirota et al. 2021) and others have developed new CR tests adding more items to the original ones (e.g., Finucane and Gullion 2010; Toplak et al. 2014; Primi et al. 2015). Recently, some researchers have shown interest in exploring whether the numerical content of the CRT affects the scores on the CR tests. In order to control the possible effects of numerical content, they developed verbal-CR tests (e.g., Sirota et al. 2021; Thomson and Oppenheimer 2016). Like CRT-3 items, these tests trigger an immediate answer and though they might involve numbers in their statements, mathematical operations are not required to find the correct answer.

A number of studies on the CRT-3 performance have found differences regarding sex on CRT scores. It has been observed that men tend to score higher than women on the test. These differences seem to be present across samples, countries, and types of CR tests (Brañas-Garza et al. 2019; Brosnan et al. 2014; Campitelli and Gerrans 2014; Frederick 2005; Nieuwenstein and van Rijn 2012; Primi et al. 2018; Razmyar and Reeve 2013; Sirota et al. 2021; Yilmaz and Saribay 2016; Toplak et al. 2017). Although the literature on the mechanisms that could explain the sex differences on CRT scores is scarce, the most widespread conjecture is that these differences could be related to the numerical content of the CRT. As it was previously mentioned, the CRT assesses the CR using arithmetical problems. This suggests that numerical ability could explain some variance on CR scores. Previous studies have found evidence that supports this fact (Avram 2018; Erceg et al. 2019; Morsanyi et al. 2017; Otero et al. 2022; Poore et al. 2014; Primi et al. 2015; Welsh et al. 2013). For instance, a recent meta-analysis conducted by Otero et al. (2022) reported that the best estimation of the relationship between CR and numerical ability is 0.62. Although some meta-analytic studies did not support the existence of sex differences on mathematic achievement (see, Else-Quest et al. 2010; Lindberg et al. 2010), some meta-analytic studies have shown that women tend to experience more anxiety doing mathematic tasks, and they tend to feel less comfortable and confident regarding their math ability. These findings were cross-culturally replicated (Else-Quest et al. 2010; Hyde et al. 1990). Congruously, some studies have found a negative relationship between math anxiety and CR scores (Morsanyi et al. 2014; Primi et al. 2017, 2018; Skagerlund et al. 2018), with the effects of anxiety on CR scores being directly and indirectly through mathematical knowledge. A positive relationship between CR scores and participants’ perceptions of their numerical abilities has also been found (Liberali et al. 2012; Primi et al. 2015; Zhang et al. 2016). According to these findings, several researchers have suggested that the numerical content of the CRT (either through numerical ability, math knowledge, math anxiety, or subjective perceptions) could explain the differences between men and women in CR scores. In order to control for the effects of numerical content, verbal-CR tests were developed (see, Sirota et al. 2021; Thomson and Oppenheimer 2016). Previous studies have not found significant differences between men and women on CR scores when verbal-CR tests were used (Bar-Hillel et al. 2019; Bronstein et al. 2019; Byrd and Conway 2019; Yilmaz and Saribay 2017). Therefore, the type of CR tests could be moderating the sex differences on CR.

Scientifically, it seems relevant to meta-analytically estimate the magnitude of sex differences on CR since CR tests scores are associated with many aspects of everyday life. For instance, people who score higher on CR tests show less risk aversion and greater patience of recompense return (*r* ranges from 0.10 to 0.29; Campitelli and Labollita 2010; Cokely and Kelley 2009; Frederick 2005); they use fewer shortcuts making decisions and judgments (the magnitude of the effect size varied according to the heuristic; Hoppe and Kusterer 2011; Moritz et al. 2014; Sirota and Juanchich 2011; Toplak et al. 2011, 2014); they are more resistant to stereotypes and prejudices (Lubian and Untertrifaller 2013); they have better experience of humor (*r* = 0.35; Ventis 2015); they tend to hold fewer religious and paranormal beliefs (*r* ranges from −0.15 to −0.33; Cheyne and Pennycook 2013; Pennycook et al. 2012; Shenhav et al. 2012); they show more subjective well-being (*r* = 0.13; Lado et al. 2021); they score higher on cognitive abilities tests (*p* ranges from 0.53 to 0.79; Otero 2019; Otero and Alonso 2023; Otero et al. 2022); and they show higher results on training proficiency and job performance (*p* ranges from 0.31 to 0.37: and 0.32 to 0.36, respectively; Otero et al. 2021; Salgado et al. 2019; Toplak et al. 2014), among others. 

Accordingly, exploring the differences between men and women in CR tests becomes a relevant matter for different disciplines (e.g., economy, organizational psychology, sociology, theology). To the best of our knowledge, four meta-analyses have been performed up to now to test the sex differences on CR (i.e., Brañas-Garza et al. 2019; Cueva et al. 2016; Primi et al. 2018; Sirota et al. 2021). All of them have reported differences between both groups (i.e., men and women) on CR, with these being in favor of men. For instance, Cueva et al. (2016) reported statistically significant differences between men and women on CRT scores (1.12 vs. 0.58: respectively, *p* < 0.001). Primi et al. (2018) found an observed effect size of d = 0.53: and Sirota et al. (2021) found an observed Hedges’ G coefficient of 0.29. Despite the contributions of these meta-analyses, new quantitative integration is still needed because of the following reasons. First, the meta-analyses of Cueva et al. (2016), Primi et al. (2018), and Sirota et al. (2021) were carried out without doing an exhaustive literature review. These meta-analyses include a few studies developed by a reduced group of researchers. Hence, the number of samples integrated were small and the sampling error is still affecting the results. Respectively, the meta-analyses include 8 (*N* = 1180), 13 (*N* = 2536), and 5 (*N* = 1012) samples. Second, the meta-analyses of Cueva et al. (2016) and Brañas-Garza et al. (2019) do not report an effect size of the sex differences on CR nor do they report data to estimate them. Finally, none of the four meta-analyses correct their results by other artifactual errors (e.g., measurement error) than the sampling error. The best estimator of the true effect size is the one which the observed effect size has been corrected by using all possible sources of error (Schmidt and Hunter 2015). Therefore, as a whole, these issues warrant the development of a new meta-analysis of the sex differences on CR which expands the results of previous meta-analyses. 

Therefore, the current article aims to contribute to the CR literature by examining the sex differences in CRT scores. The cumulation of knowledge from the results of many studies (i.e., the quantitative integration or meta-analysis) is the best method to establish robust facts and to obtain faithful estimates of the population. Hence, we aim to provide an estimate of the population average effect size across studies that examines the sex differences in CR using the psychometric meta-analysis with artifactual corrections. We also aim to explore the sex differences in CR according to the type of CR tests: verbal-CR test and numerical-CR test (i.e., 3-item CRT and larger tests) in order to determine whether the CR test type moderates the sex differences.

## 2. Methods

### 2.1. Literature Search

A literature search was conducted to identify published and unpublished studies related to CRT between September 2005 and January 2020. With this purpose, several strategies were used. First, an electronic search in the ERIC database and in Google and Google Scholar meta-databases was performed. In this search, we used the keywords of “Cognitive Reflection” and “Cognitive Reflection Test”. Second, an article-by-article search was conducted in the following scientific journals: *Applied Cognitive Psychology, Cognition, Cognitive Science, Frontiers in Psychology, Journal in Applied Research, Journal of Behavioral Decision Making, Journal of Economic Behavior and Operation, Journal of Experimental Psychology: General, Journal of Operations Management, Judgment and Decision Making, Memory and Cognition, Mind and Society, Production and Operations Management, The Journal of Economic Perspectives, The Journal of Socio-Economics* (from 2005 to 2014), *Journal of Behavioral and Experimental Economics* (from 2014), and *Thinking and Reasoning*. Third, the sources cited in the references section of CR papers were also reviewed to identify additional articles. Last, researchers on the topic were contacted by email in order to obtain new studies of CR or supplementary information of the reviewed papers.

### 2.2. Inclusion Criteria and Decision Rules

**Figure 1:**
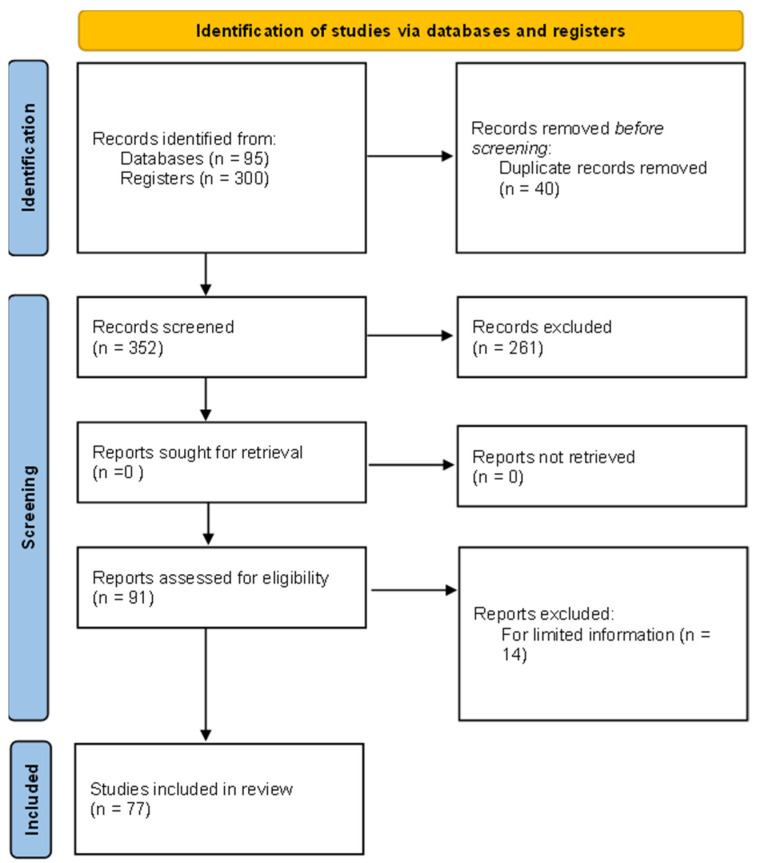
PRISMA flowchart of information through the different phases of a systematic review.

Overall, 95 records through database searching and 300 additional records through other strategies were identified. The content of each paper was examined to determine its inclusion in the analyses. To be included, the study had to provide an indicator of the sex differences in CR or other information that allowed us to estimate an effect size. Some primary studies on the relationship between CR and sex were excluded because (1) they did not empirically test the existence of differences in CR scores between men and women, or (2) the data reported were insufficient to estimate an effect size (e.g., Ibanez et al. 2013; Corgnet et al. 2015b). Likewise, we excluded those studies where the estimation of sex differences in the CRT was confusing or did not allow us to make a clear interpretation (e.g., Nieuwenstein and van Rijn 2012). We also excluded the studies that established a time limit to take the CRT (e.g., Ring et al. 2016), as this requirement might add an additional error to our findings and inflate the true variability. Therefore, the meta-analysis was conducted with a final database of 77 documents. The PRISMA flowchart (Page et al. 2021) of the literature review can be seen in Figure 1.

The meta-analyses included studies in which numerical and verbal-CR tests were used to assess CR. The numerical-CR tests included were (1) the CRT-3 (Frederick 2005; Kahneman 2011; Kahneman and Frederick 2002, 2005), (2) extended versions of the CRT-3 (i.e., the original items plus new numerical items), and (3) CR tests consisting entirely of new numerical items. The length range of longer numerical-CR tests was from 4 to 11 items (for more details, see the third column of Table S1 of the Supplementary Materials). The category of verbal-CR tests included only tests composed exclusively of verbal items. The length range of verbal-CR tests was from 3 to 10 items (for more details, see the third column of Table S1 of the Supplementary Materials).

Every meta-analysis integrated one single effect size per sample. However, primary research often reports more than one effect size (e.g., for different CR tests) for the same sample. In those cases, in which a general CRT-3 effect size was provided together with more specific CR effect sizes (i.e., other numerical-CR tests and verbal-CR tests), the first was preferred and thus integrated for the main meta-analysis. The specific results for the other types of CR tests (i.e., verbal-CR test and other numerical-CR tests) were considered for the moderator analyses. The CR tests composed of verbal items were examined separately from the numerical-CR tests to verify whether this type of test controls for the sex effects the scores. The effect sizes that were provided from CR tests composed of a combination of verbal and numerical items were excluded from this detailed analysis (e.g., the study 2 from CRT-13 of Białek et al. 2019; Böckenholt 2012; Broyd et al. 2019).

One effect size was integrated per sample. Therefore, in those cases in which the studies reported an effect size for the CRT-3 and another effect size for other numerical-CR tests for the same sample, the obtained effect size from the CRT-3 was integrated. Afterwards, the sex differences were examined by exploring the type of numerical test (i.e., CRT-3 and other numerical-CR tests) as a moderator variable. The CR tests composed of verbal items were examined separately from the numerical-CR tests to verify whether this type of test controls for the sex effects on the scores. The effect sizes that were obtained from CR tests composed of a combination of verbal and numerical items were excluded from this investigation (e.g., study 2 from CRT-13 of Białek et al. 2019; Böckenholt 2012; Broyd et al. 2019).

In the search procedure, four meta-analyses about sex differences on the CRT were found (i.e., Cueva et al. 2016; Brañas-Garza et al. 2019; Primi et al. 2018; Sirota et al. 2021). The meta-analytic results of Cueva et al. (2016) and Brañas-Garza et al. (2019) were not integrated into this study due to a lack of information estimating the effect sizes. Instead, we integrated the primary studies included in those meta-analyses to which we had access. The meta-analyses of Primi et al. (2018) and Sirota et al. (2021) were included in our study given the fact that (1) they reported data to be included and (2) we did not have full access to all primary studies. 

In order to represent the variability of these meta-analyses in our results, we developed an empirical distribution of *δ* for each meta-analysis and the values of these distributions were integrated in our analyses (see Table S1 of Supplementary Materials). The effect size *δ* is the mean effect size corrected for artifactual errors (in these cases, only by sampling error). It is interpreted as the differences between the means in the standard score form (Schmidt and Hunter 2015). Regarding Primi et al.’s meta-analysis (2018), the empirical distribution was estimated from the following information: *δ* = 0.529: *SD_δ_* = 0.095: *CI 95%* (LL = 0.34: UL = 0.72), *K* = 13: and *N* = 2536. Regarding Sirota et al.´s meta-analysis (2021); the empirical distribution was calculated from the following information: Hedges’ G = 0.29: *SD_G_* = 0.065: *CI 95%* (LL = 0.16: UL = 0.42), *K* = 5: and *N* = 1012. This study also reported the sex differences regarding verbal-CR tests. Hence, a *δ* distribution for a verbal-CR test was also developed for this sample. The data used to estimate the distribution were: Hedges´ G = −0.06: *SD_G_* = 0.07: *CI 95%* (LL = −0.20: UL = 0.07), *K* = 5: and *N* = 1012. 

Finally, the direction of the effect sizes was checked in order to unify their signs according to the following codification rule: 1 = men and 0 = women. Therefore, positive effect sizes indicate that men score higher than women on CR and negative effect sizes indicate that women score higher than men.

On this basis, the meta-analysis of the sex differences on numerical-CR tests was conducted with an accumulated sample size of 66,109 subjects and 112 effect sizes. However, when the meta-analysis was developed using only the CRT-3 the accumulated sample size was composed of 59,822 subjects and the number effect sizes integrated was 89. When using other numerical-CR tests, larger than CRT-3, the meta-analysis was conducted with an accumulated sample size of 11,511 subjects and 31 effect sizes. Last, the accumulated sample size integrated in the meta-analysis of verbal-CR tests was composed of 9916 subjects and 25 effect sizes were included. According to the MARS and the PRISMA guidelines, the primary studies included in the meta-analyses and the relevant information about them (i.e., sample size, observed effect size, measurement error in the dependent variable, and the type of CR test) can be found in a file of Supplementary Materials.

### 2.3. Meta-Analytic Method

We conducted a psychometric meta-analysis using the software package developed by Schmidt and Le (2004) based on the Schmidt and Hunter (2015) meta-analysis methods. These methods estimate the amount of observed variance (in findings across studies) due to artifactual errors. The artifacts controlled in the current meta-analysis were sampling error and measurement error in the dependent variable. Studies rarely provide all the information required to individually correct the observed effect sizes. For this reason, we developed an empirical distribution of measurement reliability, and then we corrected the average observed effect size (*d*) for this artifact to obtain the corrected effect size (*δ*).

Four reliability distributions of the dependent variable were developed, one for every meta-analysis (i.e., numerical-CR tests, CRT-3: other numerical-CR tests, and verbal-CR tests). They were created using the internal consistency coefficients reported in the primary studies. The mean and the standard deviation of the reliability distributions appear on Table 1.

Following the Schmidt and Hunter (2015) recommendations, we reported in our study the following statistics: (1) *K*, that is, the number of independent samples integrated in the meta-analysis. It is desirable that *K* be as large as possible, because the results will be less affected by sampling errors. (2) *N*, that is, the total sample size integrated on the meta-analysis. *N* should be also as larger as possible to minimize the effects of sampling errors in the results. (3) *d_w_* is the average observed effect size weighted by the study sample size. It is the effect size corrected only by the sampling errors. The larger the *d_w_* indicates the greater the sex differences between men and women. (4) *SD_d_,* which is the standard deviation of *d_w_,* indicates the variability of *d* values across studies. (5) *δ* is the corrected effect size, that is, the average effect size corrected using the sampling error, and measurement error on dependent variables. The larger of *δ* indicates the greater the sex differences between men and women on the population. (6) *SD_δ_* is the standard deviation of *δ*. *SD_δ_* indicates the variability of *δ* values across studies. (7) %VE is the percentage of observed variance explained by artifacts of sampling errors and measurement errors in the dependent variable. If %VE is high, it indicates that a larger proportion of observed variance in *d* values across studies are due to artifactual errors, hence it would not be real variability. (8) *90% CV* is the 90% credibility value. In our study, if *90% CV* is zero or negative, it would be indicated that the findings are not generalizable to other potential studies, and (9) *95% CI* is the 95% confidence intervals of *δ*. It is desirable that *95% CI* does not include the zero value, which would mean that the *δ* value is statically different from zero.

## 3. Results

The meta-analytic results on the differences between men and women in CR are shown in Table 2. The results of the meta-analysis conducted with numerical-CR tests appear in the first row. The results exploring the type of numerical-CR tests (i.e., CRT-3 or other numerical-CR tests) as a moderator appear in the second and third rows. Finally, in the last row, the results of the meta-analysis conducted with verbal-CR tests are shown.

From left to right, the columns of the table report (1) the number of independent samples integrated in the meta-analysis (*K*); (b) the total sample size (*N*); (3) the average observed effect size weighted by the study sample size (*d_w_*); (4) the standard deviation of *d_w_* (*SD_d_*); (5) the corrected effect size (*δ*); (6) the standard deviation of *δ* (*SD_δ_*); (7) the percentage of observed variance explained by artifacts (i.e., sampling error and measurement error in the dependent variable; *%VE*); (8) the 90% credibility value (*90% CV*); and (9) the 95% confidence intervals of *δ* (*95% CI*).

The meta-analytic results of the numerical-CR tests show that men scored higher than women in CR. The observed effect size and the corrected effect sizes were 0.39 and 0.47, respectively. The values indicate that the magnitude of the sex differences is small (Cohen 1977). Sampling error and CR reliability explained 36.91% of the observed variance, which means that other variables could be moderating the sex differences in CR. The 90% credibility value is different from zero, which indicates that the findings are generalizable to the population.

Therefore, the first meta-analysis permits us to conclude that, on average, men score almost half a standard deviation more than women in numerical-CR tests, and that these differences are generalizable across samples and numerical-CR measurements. 

The type of numerical-CR test was explored as a possible moderator of the sex differences on CR. Thus, the studies were classified into two categories: (1) one category composed of the studies that used the CRT-3 of Frederick (2005; see also Kahneman 2011; Kahneman and Frederick 2002, 2005) to assess CR, and (2) another category composed of the studies that used larger numerical-CR tests (more than the three original items) to assess CR. The results of these analyses show that men scored higher than women in CR in both types of numerical-CR tests. The observed effect size and the corrected effect size were 0.39 and 0.47, respectively, for CRT-3 and 0.45 and 0.52 for other numerical-CR tests. The observed and the corrected effect sizes of the CRT-3 were slightly lower than their respective values for the other numerical-CR tests. Moreover, the 95% confidence intervals almost completely overlap for both types of numerical-CRT. Hence, these findings indicate that the type of numerical-CR measurement did not affect the sex differences in CR. 

The results using verbal-CR tests also show differences between men and women in CR, but these differences were smaller than for the case of the numerical-CR tests. The meta-analytic results report an observed effect size of 0.10 and a corrected effect size of 0.13. The lower and upper bounds of the 95% confidence interval were positive, which means that the population effect size was different from zero. However, the 90% credibility value included zero, which means that the finding is not generalizable to other potential studies (Schmidt and Hunter 2015; Whitener 1990). 

Finally, the percentage of variability (i.e., observed variance) explained by artifactual errors (i.e., sampling error and measurement error on CR) was of 32.91% in CRT-3, but this percentage was substantially higher in other numerical-CR tests (60.37%) and verbal-CR-tests (60.28%), which suggests that other variables could be moderating the sex differences in CRT-3, but perhaps not in other CRT types (Schmidt and Hunter 2015).

## 4. Discussion

The study of the cognitive reflection (CR) construct has gained increasing interest in recent years. A recent search of Google Scholar indicates that there are around 5,340,000 entries with the label “Cognitive Reflection”, and Wikipedia also has an entry for the cognitive reflection test (CRT). That research points to the relevance of this construct, showing that CR is associated with very different aspects of everyday life. Thus, higher scores in CR tests are associated with a lower risk aversion and a higher patience of recompense return. Also, higher scores in CR tests are associated with fewer religious beliefs, lower use of cognitive shortcuts, higher subjective well-being, higher cognitive abilities, and better outcomes in training proficiency and job performance, among others (Cheyne and Pennycook 2013; Frederick 2005; Lado et al. 2021; Otero et al. 2021, 2022; Salgado et al. 2019; Toplak et al. 2011, 2014). 

An interesting finding regarding CR is that men tend to score higher than women in CR tests. Previous meta-analyses examining the sex differences in CR (e.g., Brañas-Garza et al. 2019; Cueva et al. 2016; Primi et al. 2018; Sirota et al. 2021) have found differences in CR scores in favor of men. However, these meta-analyses were carried out integrating very few studies (average *K* = 8), and some of them did not report an effect size of the sex differences or data to estimate it. Also, none of these studies corrected the results by artifactual errors. Therefore, it is justified to carry out a new meta-analysis to update the findings of the sex differences in CR. 

Moreover, previous studies have suggested that the differences between men and women in CR tests could be due to the mathematical content of CR tests. Hence, verbal-CR tests have been developed in order to control the mathematical content of the items. Nevertheless, the moderating effects of the CR-test type (numerical and verbal tests) on the sex differences in CR was not previously meta-analytically examined. 

Therefore, the purpose of this study was twofold. On the one hand, we aimed to conduct a meta-analytic review of the sex differences in CR. On the other hand, we aimed to explore whether the type of CR test (numerical-CR tests and verbal-CR tests) moderates the sex differences in CR. To this extent, this research has made three contributions to the literature of CR. The first one has been to show that men score higher than women in CR, although the magnitude of these differences is small (Cohen 1977). 

The second contribution has been to show that the type of CR test moderates the sex differences in CR. The results showed that, when verbal-CR tests are used, the magnitude of the sex differences was smaller (*δ* = 0.13) than when numerical-CR tests were used (*δ* = 0.46). 

The third contribution has been to show that the length of numerical tests (i.e., number of items) do not affect the differences between men and women in CR. Despite that the results showed that the sex differences in CR are slightly higher using CR tests larger than the CRT-3, the magnitude of these differences are similar for both types of measures. 

These findings have some implications for the theory. Firstly, the fact that our results show sex differences in CR test scores seems to suggest that the processes involved in performing CR tests could be different for men and women. In this sense, Campitelli and Gerrans (2014) observed that CR scores of men reflected mathematical ability, rational thinking, and disposition toward actively open-minded thinking, while the CR scores of women reflected only mathematical ability and rational thinking. However, to the best of our knowledge, there are no further studies that have explored what CR reflects in men and women separately. Hence, new studies should be developing to explore this issue in order to explain why men and women do not achieve the same results in CR tests. 

Secondly, the fact that our results show sex differences in CR test scores is not necessarily indicative that CR will predict criteria of interest (i.e., occupational performance, training proficiency, decision-making, for instance) that is significantly different for men and women. So, we must distinguish the differential validity of the CR tests to their differential prediction. The first concept refers to a situation where a test is predictive for all groups (men and women) but to different degrees; while differential prediction refers to a situation where the best prediction equations are different for both groups (Roth et al. 2014; Young 2001). To the best of our knowledge, there are no studies that have explored the differential prediction in CR tests (and according to CR test type) across men and women. Hence, it would be crucial to develop new studies for exploring this issue in order to determine whether the sex differences in CR have an impact on real outcomes. 

Thirdly, some previous studies have shown that different types of CR tests (i.e., CRT-3: larger numerical-CR tests, and verbal-CR tests) are substantially correlated. The degree of overlap suggests that different types of CR reflect the same construct (Otero 2019; Otero et al. 2022; Patel 2017; Pennycook et al. 2016; Sirota et al. 2021; Ståhl and Van Prooijen 2018; Szaszi et al. 2017; Thomson and Oppenheimer 2016; Toplak et al. 2014; among others). However, our findings show sex differences in numerical-CR tests (CRT-3 and larger CR tests) but not on verbal-CR tests. Hence, this could suggest that differences between men and women are not real sex differences, but due to some characteristics of the CR test type (e.g., numerical content). In this sense, self-image based on confidence differences in numerical tasks may be an underestimated source of variance, and this could be distorting models of system 2 performance characteristics. Consequently, it would be suitable to develop new primary studies for exploring whether self-imaging (i.e., math anxiety, the perception of math ability, etc.) has effects on performing CR tests in men and women. Also, it should be explored whether women who do not experience math anxiety (or feel confident in their math ability) perform better on CR tasks than women who experience math anxiety (or feel less confident in their math ability).

These findings also have some implications for researchers and practitioners in any field of study where the administration of CR tests could be useful. Firstly, the researchers and practitioners need to be aware that there are differences between men and women in the CR scores before the administration of the tests, particularly, when the CR tests are taken for decision-making purposes (e.g., personnel selection practices, academic admissions, or other competitive procedures). In these cases, we suggest using verbal-CR tests to minimize the sex differences in scores.

Secondly, in those cases in which numerical-CR tests are used (e.g., applied procedures with samples composed entirely by men), both the CRT-3 and the larger CR tests can be administrated. Nevertheless, we suggest using larger CR tests over CRT-3 since previous studies have shown that larger numerical-CR tests have better psychometric properties than CRT-3 (for more details, see Otero 2019; Otero et al. 2022; Primi et al. 2015; Salgado et al. 2019; Weller et al. 2013).

Finally, the present study has some limitations that should be considered. The first limitation is that the mean observed effect sizes obtained in these meta-analyses were corrected using sampling error and measurement error on the dependent variable but not for range restriction. The best estimator of the true effect size is an estimator that has been corrected using every possible source of error. Therefore, future studies should include this artifactual correction. Also, developing new primary studies about verbal-CR tests is suggested to expand the current meta-analysis and its results. We also suggest carrying out studies exploring whether numerical variables (i.e., numerical ability, math anxiety, math knowledge, and perceptions of numerical ability) mediate the relationship of CR and sex differences in different types of samples (e.g., nationality, age, education level, etc.) and CR tests (i.e., numerical and verbal CR tests).

## 5. Conclusions

In summary, this research has shown that men score higher than women in CR, although the magnitude of these differences is small. The findings also show that the type of CR test (i.e., numerical and verbal tests) moderates the sex differences, with these being larger in numerical-CR tests. Finally, the results have also suggested that the length of numerical tests (i.e., number of items) did not affect the differences between men and women in CR.

## Figures and Tables

**Table 1:** Reliability distribution of CR tests.

	*K*	${\bar{r}}_{xx}$	*SD*	Min.–Max.
Numerical-CR tests	53	0.70	0.088	0.43–0.85
CRT-3	46	0.68	0.085	0.43–0.80
Other numerical-CR tests	13	0.75	0.064	0.65–0.85
Verbal-CR tests	15	0.60	0.089	0.45–0.83

Note. *K* = number of cases; ${\bar{r}}_{xx}$ = average internal consistency reliability; *SD* = the standard deviation of *r*_xx_; Min.–Max. = minimum and maximum value of *r*_xx_*;* CR = cognitive reflection; CRT-3 = cognitive reflection test of Frederick (2005).

**Table 2:** Meta-analytic results of the sex differences in CR tests.

	Meta-Analysis of Observed Effect Size					Meta-Analysis of Corrected Effect Size				
	*K*	*N*	*d_w_*	*SD_d_*		*δ*	*SD_δ_*	*%VE*	90% *CV*	95% *CI_δ_*
Numerical-CR tests	112	66,109	0.39	0.143		0.47	0.137	36.91	0.29	0.44/0.50
CRT-3	89	59,822	0.39	0.142		0.47	0.142	32.91	0.29	0.43/0.50
Other numerical CR tests	31	11,511	0.45	0.138		0.52	0.100	60.37	0.40	0.47/0.58
Verbal-CR tests	25	9916	0.10	0.130		0.13	0.106	60.28	−0.01	0.06/0.19

Note. Positive effect sizes mean that men score higher in CRT than women. *K* = number of independent samples, *N* = sample size; *d_w_* = the average observed effect size weighted by the study sample size; *SD_d_* = the standard deviation of *d_w_*; *δ* = corrected effect size; *SD_δ_* = the standard deviation of *δ*; %*VE* = the percentage of observed variance explained by all artifactual errors; 90% *CV* = the 90% credibility value; 95% *CI_δ_* = the 95% confidence intervals of *δ*; CRT-3 = cognitive reflection test of Frederick (2005); other-CR tests = other numerical-CR tests different from the CRT-3.

## Data Availability

The dataset used and/or analyzed during this study is available in Supplementary File.

## Supplementary Materials

The following supporting information can be downloaded at: https://www.mdpi.com/article/10.3390/jintelligence12040039/s1, Table S1: Studies Included in the Meta-analysis of the Sex Differences on CR. (Aczel et al. 2015; Aktas et al. 2017; Albaity et al. 2014; Alós-Ferrer and Hügelschäfer 2012; Alós-Ferrer et al. 2016; Baron et al. 2015; Bosch-Domènech et al. 2014; Bosley et al. 2019; Browne et al. 2014; Burger et al. 2020; Cáceres and Martín 2017; Calvillo et al. 2020; Capraro et al. 2017; Čavojová et al. 2019; Cheng and Janssen 2019; Corgnet et al. 2015a, 2016; Drummond and Fischhoff 2017; Duttle and Inukai 2015; Fosgaard et al. 2019; Gervais et al. 2018; Grossman et al. 2014; Guthrie et al. 2007; Kahan 2017; Kiss et al. 2016; Koehler and Pennycook 2019; Lohse 2016; Mandel and Kapler 2018; Muñoz-Murillo et al. 2020; Narayanan and Moritz 2015; Obrecht et al. 2009; Otero 2020; Pennycook and Rand 2019a, 2019b; Ponti and Carbone 2009; Ponti et al. 2014; Royzman et al. 2015; Sajid and Li 2019; Schulze and Newell 2015; Sinayev and Peters 2015; Šrol 2018; Stagnaro et al. 2018; Stagnaro et al. 2019; Stieger and Reips 2016; Svenson et al. 2018; Teigen et al. 2018; Willard and Norenzayan 2017; Woike 2019; Yilmaz et al. 2020) are cited in the supplementary materials.
