# Supplementary material for: Sex Differences in Cognitive Reflection: A Meta-Analysis
Source: J Intell. 2024 Mar 29;12(4):39. doi: 10.3390/jintelligence12040039 (PMC11051326; doi:10.3390/jintelligence12040039)
Supplement: Supplementary file 1 [file jintelligence-12-00039-s001.zip › jintelligence-2786099-supplementary.pdf]

Supplementary Materials of the Manuscript:

Sex Differences on Cognitive Reflection: A meta-analysis

Inmaculada Otero, Alexandra Martínez, Dámaris Cuadrado, Mario Lado, Silvia Moscoso, and Jesús F. Salgado

Table S1.

Studies Included in the Meta-analysis of the Sex Differences on CR

Study	Year	Type of CRT	*N*	d_{xy}	r_{xx}	Sample
Aczel et al.	2015	original	864	0.256	.62	
Aktas et al.	2017	original	267	0.124	.54	Study 1
		original	239	0.345	.63	Study 2
		original	382	-0.165	.73	Study 3
Albaity et al.	2014	original	880	0.486	-	
Alós-Ferrer et al.	2016	original	155	0.476	-	
Alós-Ferrer & Hügelschäfer	2012	original	416	0.827	-	Exp. 1
		original	111	0.528	-	Exp. 2
		original	364	0.335	-	Exp. Class
Avram	2018	original	194	0.441	-	
Bar-Hillel et al.	2019	V-CRT	394	0.056	.49	
Baron et al.	2015	original	103	0.242	.62	
Bialek et al.	2019	CRT-6	123	0.258	.78	Study 1
		original	122	0.124	.78	Study 2
Bosch-Doménech et al.	2014	original	623	0.394	-	
Bosley et al.	2019	original	451	0.438	.62	
Bronstein et al.	2019	original	947	0.387	.70	
		V-CRT	947	0.068	.63	
Brosnan et al.	2014	original	68	0.517	-	
Browne et al.	2014	original	1,137	0.538	-	
Burger et al.	2020	original	399	0.266	.54	Study 1
		CRT-6	399	0.322	.78	Study 1
		original	304	0.458	.53	Study 2
		CRT-6	304	0.516	.77	Study 2
Byrd & Conway	2019	original	276	0.181	.80	Study 1
		V-CRT	276	-0.080	-	Study 1
		original	191	0.120	.78	Study 2
		V-CRT	191	0.100	-	Study 2
Cáceres & San Martín	2017	original	19	1.060	-	
Calvillo et al.	2019	CRT-7	411	0.539	-	Exp. 1
		CRT-7	231	0.086	-	Exp. 2
Campitelli & Gerrans	2014	original	2,019	0.336	.66	
Campitelli & Labollita	2010	original	155	0.220	-	
Capraro et al.	2017	CRT-7	677	0.604	-	
Čavojová et al.	2019	CRT-7	121	0.604	.77	
Cheng & Janssen	2019	V-CRT	135	0.110	.60	
Corgnet et al.	2015	CRT-7	150	0.603	-	Study 1
		CRT-7	158	0.673	-	Study 2

Table S1.

Continuation

Study	Year	Type of CRT	N	d_{xy}	r_{xx}	Sample
Corgnet et al.	2016	CRT-7	150	0.549	-	
Drummond & Fischhoff	2017	original	395	0.383	.80	Study 1a
		original	393	0.324	.73	Study 1c
		original	268	0.307	.80	Study 2
Duttle & Inukai	2015	original	66	1.155	-	
Finucane & Gullion	2010	CRT-6	608	0.408	.80	
Fosgaard et al.	2019	original	1,926	0.377	-	
Frederick	2005	original	3,428	0.112	-	
Gervais et al.	2018	original	3,461	0.370	-	
Grossman et al.	2014	CRT-4	224	0.346	-	
Guthrie et al.	2007	original	241	0.132	-	
Kahan	2017	original	1,993	0.381	.65	
Kiss et al.	2016	original	900	0.343	-	
Koehler & Pennycook	2019	V-CRT	866	0.138	.61	
Lohse	2016	original	284	0.359	-	
Narayanan & Moritz	2015	original	96	0.062	-	
Mandel & Kapler	2018	original	190	0.636	.71	Exp. 1
		original	316	0.223	.80	Exp. 2
Muñoz-Murillo et al.	2020	original	195	0.676	-	
Obrecht et al.	2009	original	417	0.606	-	
[a]Otero	2019	original	1,367	0.452	.58	
		CRT-10	1,367	0.632	.70	
Otero	2020	CRT-10	186	0.325	.65	Study 2
		original	186	0.113	.64	Study 2
		V-CRT	186	0.475	.45	Study 2
Pennycook et al.	2012	original	223	0.324	-	Study 1
		original	267	0.473	-	Study 2
Pennycook et al.	2016	original	369	0.510	-	
Pennycook & Rand	2019a	original	799	0.320	.68	Study 1
		V-CRT	790	0.158	.57	Study 1
		original	2,630	0.512	.71	Study 2
		V-CRT	2,615	0.150	.58	Study 2
Pennycook & Rand	2019b	original	401	0.351	.74	Study 1
		V-CRT	401	0.092	.63	Study 1
		original	397	0.233	.74	Study 2
		V-CRT	395	0.134	.54	Study 2

Table S1.

Continuation

Study	Year	Type of CRT	*N*	d_{xy}	r_{xx}	Sample
Ponti & Carbone	2009	original	48	0.873	-	
Ponti et al.	2014	original	192	0.512	-	
[a]Primi et al.	2015	original	939	0.340	-	
		CRT-6	908	0.320	.76	
Primi et al.	2018	original	281	0.719	-	Study 1b
		original	282	0.624	-	
		original	282	0.624	-	
		original	282	0.529	-	
		original	282	0.529	-	
		original	282	0.529	-	
		original	282	0.434	-	
		original	282	0.434	-	
		original	281	0.339	-	
		CRT-6	181	0.699	.76	Study 2
Razmyar & Reeve	2013	original	150	0.606	.64	
Royzman et al.	2014	original	527	0.372	.75	
Royzman et al.	2015	CRT-6	548	0.335	.85	
Sajid & Li	2019	original	601	0.508	.72	
Schulze & Newell	2015	original	58	0.817	-	Exp. 2a
		original	58	0.829	-	Exp. 2b
Sinayev & Peters	2015	CRT-5	1,459	0.473	-	Study 2
[b]Sirota et al.	2021	[c]CRT-N	112	0.160	.75	
		[c]CRT-N	113	0.225	.75	
		[c]CRT-N	113	0.225	.75	
		[c]CRT-N	113	0.290	.75	
		[c]CRT-N	113	0.290	.75	
		[c]CRT-N	113	0.290	.75	
		[c]CRT-N	113	0.355	.75	
		[c]CRT-N	113	0.355	.75	
		[c]CRT-N	111	0.420	.75	
		V-CRT	112	-0.200	.83	
		V-CRT	113	-0.130	.83	
		V-CRT	113	-0.130	.83	
		V-CRT	113	-0.060	.83	
		V-CRT	113	-0.060	.83	

Table S1.

Continuation

Study	Year	Type of CRT	*N*	d_{xy}	r_{xx}	Sample
		V-CRT	113	-0.060	.83	
		V-CRT	113	0.010	.83	
		V-CRT	113	0.010	.83	
		V-CRT	111	0.070	.83	
Skagerlund et al.	2018	original	2,058	0.432	.63	
Šrol	2019	original	1,204	0.550	.70	
		CRT-4	1,204	0.520	.65	
Stagnaro et al.	2018	original	2,759	0.364	-	
Stagnaro et al.	2019	original	513	0.279	.63	Study 1
		original	545	0.421	.69	Study 2
		V-CRT	545	0.324	.65	Study 2
Ståhl & Van Prooijen	2018	original	333	-0.098	.72	Study 1
		V-CRT	333	-0.293	.60	Study 1
		original	302	0.345	.74	Study 2
		V-CRT	302	0.060	.66	Study 2
Stieger & Reips	2016	original	2,127	0.275	.64	
Svenson et al.	2018	original	122	0.438	.69	
Teigen et al.	2018	original	130	0.570	.71	
[a]Thomson & Oppenheimer	2016	original	129	0.813	.62	
		V-CRT	131	0.248	.51	
[a]Toplak et al.	2014	original	158	0.637	-	
		CRT-4	158	0.652	-	
Toplak et al.	2017	CRT-11	232	0.473	.80	
Ventis	2015	original	146	0.449	.67	
Welsh et al.	2013	original	102	0.516	-	
Willard & Norenzayan	2017	original	1,006	0.306	-	
Woike	2019	original	698	0.250	.78	Study 2
		CRT-3	698	0.330	.66	Study 2
Yilmaz et al.	2019	original	8,647	0.445	-	
Yilmaz & Saribay	2016	original	337	0.293	.72	Study 1
		original	691	0.600	.50	Study 2
		original	126	0.128	.43	Study 3a
		original	86	0.473	.70	Study 3b
[a]Yilmaz & Saribay	2017	original	395	0.366	.75	
		V-CRT	395	-0.020	.64	
Zhang et al.	2016	Original	205	0.370	.75	

Note. N = sample size; d_{xy} = observed effect size; r_{xx} = internal consistency reliability of CR; CRT-3 = cognitive reflection test of Frederick (2005); V-CRT = verbal-CRT.
[a]The effect sizes are from the same sample but using different CR test.
[b]The distribution of effect sizes were obtained from a meta-analytic study.
[c]The CRT used in this study is a mixture of the CRT-3 (Frederick, 2005) and the CRT-7 of Toplak et al. (2014).

REFERENCES:

Aczel, B., Bago, B., Szollosi, A., Foldes, A., and Lukacs, B. 2015. Measuring individual differences in decision biases: Methodological 451 considerations. Frontiers in Psychology, 6, 1770. https://doi.org/10.3389/fpsyg.2015.01770 452

Aktaş, B., Yılmaz, O., and Bahçekapılı, H. G. 2017. Moral pluralism on the trolley tracks: Different normative principles are used for 453 different reasons in justifying moral judgments. Judgment and Decision Making, 123, 297-307. 454

Albaity, M., Rahman, M., and Shahidul, I. 2014. Cognitive reflection test and behavioral biases in Malaysia. Judgment and Decision 455 Making, 92, 148-151. 456

Alós-Ferrer, C., Garagnani, M., and Hügelschäfer, S. 2016. Cognitive reflection, decision biases, and response times. Frontiers in 457 Psychology, 7, 1402. https://doi.org/10.3389/fpsyg.2016.01402 458

Alós-Ferrer, C., and Hügelschäfer, S. 2012. Faith in intuition and behavioral biases. Journal of Economic Behavior and Organization, 841, 459 182-192. https://doi.org/10.1016/j.jebo.2012.08.004 460

Avram, L. A. 2018. Gender differences and other findings on the cognitive reflection test. Studia Universitatis Babes Bolyai-Oeconomica, 461 633, 56-67. https://doi.org/10.2478/subboec-2018-0014 462

Bar-Hillel, M., Noah, T., and Shane, F. 2019. Solving stumpers, CRT and CRAT: Are the abilities related? Judgment and Decision 463 Making, 145, 620-623. 464

Baron, J., Scott, S., Fincher, K., and Metz, S. E. 2015. Why does the cognitive reflection test sometimes predict utilitarian moral 465 judgment and other things? Journal of Applied Research in Memory and Cognition, 43, 265–284. https://doi.org/10.1016/j.jar-466 mac.2014.09.003. 467

Białek, M., Bergelt, M., Majima, Y., and Koehler, D. J. 2019. Cognitive reflection but not reinforcement sensitivity is consistently 468 associated with delay discounting of gains and losses. Journal of Neuroscience, Psychology, and Economics, 123-4, 169-183. 469 https://doi.org/10.1037/npe0000111 470

Bosch-Domènech, A., Brañas-Garza, P., and Espín, A. M. 2014. Can exposure to prenatal sex hormones 2D: 4D predict cognitive 473 reflection? Psychoneuroendocrinology, 43, 1-10. https://doi.org/10.1016/j.psyneuen.2014.01.023 474

Bosley, S. A., Bellemare, M. F., Umwali, L., and York, J. 2019. Decision-making and vulnerability in a pyramid scheme fraud. Journal 475 of Behavioral and Experimental Economics, 80, 1-13. https://doi.org/10.1016/j.socec.2019.02.011 476

Bronstein, M. V., Pennycook, G., Bear, A., Rand, D. G., and Cannon, T. D. 2019. Belief in fake news is associated with delusionality, 479 dogmatism, religious fundamentalism, and reduced analytic thinking. Journal of Applied Research in Memory and Cognition, 81, 480 108-117. https://doi.org/10.1016/j.jarmac.2018.09.005 481

Brosnan, M., Hollinworth, M., Antoniadou, K., and Lewton, M. 2014. Is empathizing intuitive and systemizing deliberative? Person-482 ality and Individual Differences, 66, 39-43. https://doi.org/10.1016/j.paid.2014.03.006 483

Browne, M., Pennycook, G., Goodwin, B., and McHenry, M. 2014. Reflective minds and open hearts: Cognitive style and personality 484 predict religiosity and spiritual thinking in a community sample. European Journal of Social Psychology, 447, 736-742. 485 https://doi.org/10.1002/ejsp.2059 486

Burger, A. M., Pfattheicher, S., and Jauch, M. 2020. The role of motivation in the association of political ideology with cognitive 489 performance. Cognition, 195, 104124. https://doi.org/10.1016/j.cognition.2019.104124 490

Byrd, N., and Conway, P. 2019. Not all who ponder count costs: Arithmetic reflection predicts utilitarian tendencies, but logical 491 reflection predicts both deontological and utilitarian tendencies. Cognition, 192, 103995. https://doi.org/10.1016/j.cogni-492 tion.2019.06.007 493

Cáceres, P., and San Martín, R. 2017. Low cognitive impulsivity is associated with better gain and loss learning in a probabilistic 494 decision-making task. Frontiers in Psychology, 8, 204. https://doi.org/10.3389/fpsyg.2017.00204 495

Calvillo, D. P., Swan, A. B., and Rutchick, A. M. 2020. Ideological belief bias with political syllogisms. Thinking and Reasoning, 262, 496 291-310. https://doi.org/10.1080/13546783.2019.1688188 497

Campitelli, G., and Gerrans, P. 2014. Does the cognitive reflection test measure cognitive reflection? A mathematical modeling ap-498 proach. Memory and Cognition, 423, 434-447. https://doi.org/10.3758/s13421-013-0367-9 499

Campitelli, G., and Labollita, M. 2010. Correlations of cognitive reflection with judgments and choices. Judgment and Decision Making, 500 53, 182-191. 501

Capraro, V., Corgnet, B., Espín, A. M., and Hernán-González, R. 2017. Deliberation favours social efficiency by making people dis-502 regard their relative shares: Evidence from USA and India. Royal Society Open Science, 42, 160605. 503 https://doi.org/10.1098/rsos.160605 504

Čavojová, V., Secară, E. C., Jurkovič, M., and Šrol, J. 2019. Reception and willingness to share pseudo-profound bullshit and their 505 relation to other epistemically suspect beliefs and cognitive ability in Slovakia and Romania. Applied Cognitive Psychology, 332, 506 299-311. https://doi.org/10.1002/acp.3486 507

Cheng, J., and Janssen, C. 2019. The relationship between an alternative form of cognitive reflection test and intertemporal choice. 508 Studia Psychologica, 612, 86-98. https://doi.org/10.21909/sp.2019.02.774 509

Corgnet, B., Espín, A. M., and Hernán-González, R. 2015. Creativity and cognitive skills among millennials: Thinking too much and 515 creating too little. Frontiers in Psychology, 7, 1626. https://doi.org/0.3389/fpsyg.2016.01626 516

Corgnet, B., Espín, A. M., and Hernán-González, R. 2016. The cognitive basis of social behavior: Cognitive reflection overrides anti-517 social but not always prosocial motives. Frontiers in Behavioral Neuroscience, 9, 287. https://doi.org/10.3389/fnbeh.2015.00287 518

Drummond, C., and Fischhoff, B. 2017. Development and validation of the scientific reasoning scale. Journal of Behavioral Decision 524 Making, 301, 26-38. https://doi.org/10.1002/bdm.1906 525

Duttle, K., and Inukai, K. 2015. Complexity aversion: Influences of cognitive abilities, culture and system of thought. Economic Bul-526 letin, 352, 846-855. 527

Finucane, M. L., and Gullion, C. M. 2010. Developing a tool for measuring the decision-making competence of older adults. Psychol-539 ogy and Aging, 252, 271-288. https://doi.org/10.1037/a0019106. 540

Fosgaard, T. R., Hansen, L. G., and Wengström, E. 2019. Cooperation, framing, and political attitudes. Journal of Economic Behavior 541 and Organization, 158, 416-427. https://doi.org/10.1016/j.jebo.2018.12.010 542

Frederick, S. 2005. Cognitive reflection and decision making. Journal of Economic Perspectives, 19, 25-42. 543 https://doi.org/10.1257/089533005775196732 544

Gervais, W. M., van Elk, M., Xygalatas, D., McKay, R. T., Aveyard, M., Buchtel, E. E., Dar-Nimrod, I., Klocova, E. K., Ramsay, J. E.; 545 Riekki, T.; Svedholm-Hakkinen, A. M., and Bulbulia, J. 2018. Analytic atheism: A cross-culturally weak and fickle phenomenon? 546 Judgment and Decision making, 133, 268-274. 547

Grossman, Z., van der Weele, J., and Andrijevik, A. 2014. A test of dual-process reasoning in charitable giving (Working Paper). 548 University of California Santa Bárbara. https://escholarship.org/uc/item/4tm617f7 549

Guthrie, C., Rachlinski, J. J., and Wistrich, A. J. 2007. Blinking on the bench: How judges decide cases. Cornell Law Review, 931, 1-44. 550

Kahan, D. M. 2017. 'Ordinary science intelligence': A science-comprehension measure for study of risk and science communication, 557 with notes on evolution and climate change. Journal of Risk Research, 208, 995-1016. 558 https://doi.org/10.1080/13669877.2016.1148067 559

Kiss, H. J., Rodriguez-Lara, I., and Rosa-García, A. 2016. Think twice before running! Bank runs and cognitive abilities. Journal of 566 Behavioral and Experimental Economics, 64, 12-19. https://doi.org/10.1016/j.socec.2015.01.006 567

Koehler, D. J., and Pennycook, G. 2019. How the public, and scientists, perceive advancement of knowledge from conflicting study 568 results. Judgment and Decision Making, 166, 671-682. 569

Lohse, J. 2016. Smart or selfish–when smart guys finish nice. Journal of Behavioral and Experimental Economics, 64, 28-40. 579 https://doi.org/10.1016/j.socec.2016.04.002 580

Mandel, D. R., and Kapler, I. V. 2018. Cognitive style and frame susceptibility in decision-making. Frontiers in Psychology, 9, 1461. 583 https://doi.org/10.3389/fpsyg.2018.01461 584

Muñoz-Murillo, M., Álvarez-Franco, P. B., and Restrepo-Tobón, D. A. 2020. The role of cognitive abilities on financial literacy: New 594 experimental evidence. Journal of Behavioral and Experimental Economics, 84, 101482. https://doi.org/10.1016/j.socec.2019.101482 595

Narayanan, A., and Moritz, B. B. 2015. Decision making and cognition in multi-echelon supply chains: An experimental study. 596 Production and Operations Management, 248, 1216-1234. https://doi.org/10.1111/poms.12343 597

Obrecht, N. A., Chapman, G. B., and Gelman, R. 2009. An encounter frequency account of how experience affects likelihood estima-600 tion. Memory and Cognition, 375, 632-643. https://doi.org/10.3758/MC.37.5.632. 601

Otero, I. 2019. Construct and criterion validity of cognitive reflection [Doctoral dissertation, University of Santiago de Compostela]. 602 https://minerva.usc.es/xmlui/handle/10347/20521 603

Otero, I. 2020. [Unpublished raw data on the sex differences in cognitive reflection]. University of Santiago de Compostela. 604

Pennycook, G., Cheyne, J. A., Koehler, D. J., and Fugelsang, J. A. 2016. Is the cognitive reflection test a measure of both reflection 616 and intuition? Behavior Research Methods, 481, 341-348. https://doi.org/10.3758/s13428-015-0576-1 617

Pennycook, G., Cheyne, J. A., Seli, P., Koehler, D. J., and Fugelsang, J. A. 2012. Analytic cognitive style predicts religious and para-618 normal belief. Cognition, 1233, 335–346. https://doi.org/10.1016/j.cognition.2012.03.003 619

Pennycook, G., and Rand, D. G. 2019a. Lazy, not biased: Susceptibility to partisan fake news is better explained by lack of reasoning 620 than by motivated reasoning. Cognition, 188, 39-50. https://doi.org/10.1016/j.cognition.2018.06.011 621

Pennycook, G., and Rand, D. G. 2019b. Who falls for fake news? The roles of bullshit receptivity, overclaiming, familiarity, and 622 analytic thinking. Journal of Personality, 882, 185-200. https://doi.org/10.1111/jopy.12476 623

Ponti, G., and Carbone, E. 2009. Positional learning with noise. Research in Economics, 634, 225-241. 624 https://doi.org/10.1016/j.rie.2009.09.002 625

Ponti, G., Rodriguez-Lara, I., and Di Cagno, D. 2014. Doing it now or later with payoff externalities: Experimental evidence on social 626 time preferences (Working Paper No. 5). http://static.luiss.it/RePEc/pdf/cesare/1401.pdf 627

Primi, C., Donati, M. A., Chiesi, F., and Morsanyi, K. 2018. Are there gender differences in cognitive reflection? Invariance and 631 differences related to mathematics. Thinking and Reasoning, 242, 258-279. https://doi.org/10.1080/13546783.2017.1387606 632

Primi, C., Morsanyi, K., Chiesi, F., Donati, M. A., and Hamilton, J. 2015. The development and testing of a new version of the cogni-633 tive reflection test applying item response theory IRT. Journal of Behavioral Decision Making, 295, 453-469. 634 https://doi.org/10.1002/bdm.1883 635

Razmyar, S., and Reeve, C. L. 2013. Individual differences in religiosity as a function of cognitive ability and cognitive style. Intelli-638 gence, 415, 667-673. https://doi.org/10.1016/j.intell.2013.09.003 639

Royzman, E. B., Landy, J. F., and Leeman, R. F. 2015. Are thoughtful people more utilitarian? CRT as a unique predictor of moral 645 minimalism in the dilemmatic context. Cognitive Science, 392, 325-352. https://doi.org/10.1111/cogs.12136 646

Sajid, M., and Li, M. C. 2019. The role of cognitive reflection in decision making: Evidence from Pakistani managers. Judgment and 647 Decision Making, 145, 591-604. 648

Schulze, C., and Newell, B. R. 2015. Compete, coordinate, and cooperate: How to exploit uncertain environments with social inter-654 action. Journal of Experimental Psychology: General, 1445, 967-981. https://doi.org/10.1037/xge0000096 655

Sinayev, A., and Peters, E. 2015. Cognitive reflection vs. calculation in decision making. Frontiers in Psychology, 6, 532. 658 https://doi.org/10.3389/fpsyg.2015.00532 659

Sirota, M., Kostovičová, L., Juanchich, M., Dewberry, C., and Marshall, A. C. 2021. Measuring cognitive reflection without maths: 662 Developing and validating the verbal cognitive reflection test. Journal of Behavioral Decision Making, 343, 322-343. 663 https://doi.org/10.1002/bdm.2213 664

Skagerlund, K., Lind, T., Strömbäck, C., Tinghög, G., and Västfjäll, D. 2018. Financial literacy and the role of numeracy–how indi-665 viduals' attitude and affinity with numbers influence financial literacy. Journal of Behavioral and Experimental Economics, 74, 18-666 25. https://doi.org/10.1016/j.socec.2018.03.004 667

Šrol, J. 2018. Dissecting the expanded cognitive reflection test: An item response theory analysis. Journal of Cognitive Psychology, 307, 673 643-655. https://doi.org/10.1080/20445911.2018.1518327 674

Stagnaro, M., Pennycook, G., and Rand, D. G. 2018. Performance on the cognitive reflection test is stable across time. Judgment and 675 Decision Making, 133, 260-267. 676

Stagnaro, M. N., Ross, R. M., Pennycook, G., and Rand, D. G. 2019. Cross-cultural support for a link between analytic thinking and 677 disbelief in God: Evidence from India and the United Kingdom. Judgment and Decision Making, 142, 179-186. 678

Ståhl, T., and Van Prooijen, J. W. 2018. Epistemic rationality: Skepticism toward unfounded beliefs requires sufficient cognitive 679 ability and motivation to be rational. Personality and Individual Differences, 122, 155-163. https://doi.org/10.1016/j.paid.2017.10.026 680

Stieger, S., and Reips, U. D. 2016. A limitation of the cognitive reflection test: Familiarity. PeerJ Life and Enviroment, 4, e2395. 682 https://doi.org/10.7717/peerj.2395 683

Svenson, O., Gonzalez, N., and Eriksson, G. 2018. Different heuristics and same bias: A spectral analysis of biased judgments and 684 individual decision rules. Judgment and Decision Making, 135, 401-412. 685

Teigen, K. H., Løhre, E., and Hohle, S. M. 2018. The boundary effect: Perceived post hoc accuracy of prediction intervals. Judgment 686 and Decision Making, 134, 309-321. 687

Thomson, K. S., and Oppenheimer, D. M. 2016. Investigating an alternate form of the cognitive reflection test. Judgment and Decision 688 Making, 111, 99-113. 689

Toplak, M. E., West, R. F., and Stanovich, K. E. 2014. Assessing miserly information processing: An expansion of the cognitive 692 reflection test. Thinking and Reasoning, 202, 147-168. https://doi.org/10.1080/13546783.2013.844729 693

Toplak, M. E., West, R. F., and Stanovich, K. E. 2017. Real-world correlates of performance on heuristics and biases tasks in a com-694 munity sample. Journal of Behavioral Decision Making, 302, 541-554. https://doi.org/10.1002/bdm.1973 695

Ventis, L. 2015. Thinking fast and slow in the experience of humor. International Journal of Humor Researh, 283, 351-373. 696 https://doi.org/10.1515/humor-2015-0070 697

Welsh, M., Burns, N., and Delfabbro, P. 2013. The Cognitive Reflection Test: How much more than numerical ability? In M. Knauff, 702 N. Sebanz, M. Pauen, and I. Wachsmuth Eds., Proceedings of the annual meeting of the Cognitive Science Society Vol. 35, pp. 1587-703 1592. Psychology Press. http://hdl.handle.net/2440/83719https://cloudfront.escholarship.org/dist/prd/con-704 tent/qt68n012fh/qt68n012fh.pdf 705

Willard, A. K., and Norenzayan, A. 2017. "Spiritual but not religious": Cognition, schizotypy, and conversion in alternative beliefs. 706 Cognition, 165, 137-146. https://doi.org/10.1016/j.cognition.2017.05.018 707

Woike, J. K. 2019. Upon repeated reflection: Consequences of frequent exposure to the cognitive reflection test for Mechanical Turk 710 participants. Frontiers in Psychology, 10, 2646. https://doi.org/10.3389/fpsyg.2019.02646 711

Yılmaz, O., and Sarıbay, S. A. 2016. An attempt to clarify the link between cognitive style and political ideology: A non-western 712 replication and extension. Judgment and Decision Making, 113, 287-300. 713

Yılmaz, O., and Sarıbay, S. A. 2017. The relationship between cognitive style and political orientation depends on the measures used. 714 Judgment and Decision Making, 122, 140-147. 715

Yilmaz, O., Saribay, S. A., and Iyer, R. 2020. Are neo-liberals more intuitive? Undetected libertarians confound the relation between 716 analytic cognitive style and economic conservatism. Current Psychology, 391, 25-32. https://doi.org/10.1007/s12144-019-0130-x 717

Zhang, D. C., Highhouse, S., and Rada, T. B. 2016. Explaining sex differences on the Cognitive Reflection Test. Personality and Indi-720 vidual Differences, 101, 425-427. https://doi.org/10.1016/j.paid.2016.06.034 721
